# Placenta growth factor and vascular endothelial growth factor B expression in the hypoxic lung

**DOI:** 10.1186/1465-9921-12-17

**Published:** 2011-01-25

**Authors:** Michelle Sands, Katherine Howell, Christine M Costello, Paul McLoughlin

**Affiliations:** 1 School of Medicine and Medical Science, Conway Institute of Biomolecular and Biomedical Science, University College Dublin, Belfield, Dublin 4, Ireland

## Abstract

**Background:**

Chronic alveolar hypoxia, due to residence at high altitude or chronic obstructive lung diseases, leads to pulmonary hypertension, which may be further complicated by right heart failure, increasing morbidity and mortality. In the non-diseased lung, angiogenesis occurs in chronic hypoxia and may act in a protective, adaptive manner. To date, little is known about the behaviour of individual vascular endothelial growth factor (VEGF) family ligands in hypoxia-induced pulmonary angiogenesis. The aim of this study was to examine the expression of placenta growth factor (PlGF) and VEGFB during the development of hypoxic pulmonary angiogenesis and their functional effects on the pulmonary endothelium.

**Methods:**

Male Sprague Dawley rats were exposed to conditions of normoxia (21% O_2_) or hypoxia (10% O_2_) for 1-21 days. Stereological analysis of vascular structure, real-time PCR analysis of vascular endothelial growth factor A (VEGFA), VEGFB, placenta growth factor (PlGF), VEGF receptor 1 (VEGFR1) and VEGFR2, immunohistochemistry and western blots were completed. The effects of VEGF ligands on human pulmonary microvascular endothelial cells were determined using a wound-healing assay.

**Results:**

Typical vascular remodelling and angiogenesis were observed in the hypoxic lung. PlGF and VEGFB mRNA expression were significantly increased in the hypoxic lung. Immunohistochemical analysis showed reduced expression of VEGFB protein in hypoxia although PlGF protein was unchanged. The expression of VEGFA mRNA and protein was unchanged. *In vitro *PlGF at high concentration mimicked the wound-healing actions of VEGFA on pulmonary microvascular endothelial monolayers. Low concentrations of PlGF potentiated the wound-healing actions of VEGFA while higher concentrations of PlGF were without this effect. VEGFB inhibited the wound-healing actions of VEGFA while VEGFB and PlGF together were mutually antagonistic.

**Conclusions:**

VEGFB and PlGF can either inhibit or potentiate the actions of VEGFA, depending on their relative concentrations, which change in the hypoxic lung. Thus their actions *in vivo *depend on their specific concentrations within the microenvironment of the alveolar wall during the course of adaptation to pulmonary hypoxia.

## Background

Pulmonary hypertension frequently occurs in people suffering from hypoxic lung diseases such as chronic obstructive pulmonary disease (COPD), emphysema, cystic fibrosis and fibrosing alveolitis, often resulting in right heart failure and increased morbidity and mortality [1-3]. Such diseases cause tissue destruction within the airways and gas exchange regions of the lung, accompanied by a loss of the pulmonary vasculature (rarefaction). In addition, sustained vasoconstriction associated with thickening of the vessel wall results in lumen reduction that, together with a loss of vessels, results in increased pulmonary vascular resistance and pulmonary hypertension (PH).

Chronic alveolar hypoxia is an important mediator of the development of PH. Hypoxia in the absence of lung disease causes PH that is associated with arteriolar remodelling and increased vasoconstriction [4]. In contrast to lung disease, hypoxia alone has been shown to stimulate angiogenesis within the pulmonary circulation, demonstrating that the adult pulmonary circulation is capable of increasing the area of its gas exchange region [4,5]. Such an increase could potentially be beneficial in the adaptation to an hypoxic environment. The mechanisms underlying this are poorly understood but are of interest given the therapeutic potential in diseases that are characterised by vessel loss; e.g. idiopathic pulmonary fibrosis, pulmonary arterial hypertension and emphysema.

There is evidence to suggest that the vascular endothelial growth factor (VEGF) family has a very important role to play in the development of PH. Exposure to chronic hypoxia, with concurrent blockade of VEGFR1 and R2, results in a severe and irreversible form of PH and the development of vascular lesions similar to those observed in patients with pulmonary arterial hypertension. Moreover, upon return to a normoxic environment, the disease continues to progress and is fatal [6-8]. Conversely, overexpression of VEGFA has been shown to have protective effects in a chronic hypoxic animal model of PH. This protective effect is evidenced by a decrease in pulmonary arterial pressure and RV hypertrophy and a decrease in the percentage of muscularised arterioles [9]. These results indicate that vascular endothelial growth factors have an important role to play in ameliorating the progression of PH. However, it is currently not known which VEGF ligands are important or how these ligands interact with each other.

While the role of certain angiogenic growth factors is well characterised in the systemic circulation, to date little is known about the growth factors involved in angiogenesis within the pulmonary circulation. The VEGF family of angiogenic ligands, comprised of VEGFA, B, C, D, E and placenta growth factor (PlGF), are among the best characterised angiogenic growth factors [10]. VEGFA, B and PlGF have well-established roles in angiogenesis within the systemic vasculature, while VEGFC and D are primarily involved in lymphangiogenesis [11]. VEGFE, or viral VEGF, is found in several *orf *viral strains and *pseudocowpox *virus but is only expressed in endothelial cells of pustules following viral infection [12]. The biological effects of VEGFA, VEGFB and PlGF are mediated via two specific cell surface-expressed receptors: VEGFR1 (Flt-1) and VEGFR2 (KDR or Flk-1). Two co-receptors, neuropilin-1 (NP1) and neuropilin-2 (NP2), are known to act together with VEGFR2 to enhance signalling [13]. The system is further modulated by expression of VEGF splice variants that are inhibitors of VEGFR signalling [14,15].

To date, little is known of the biological functions of PlGF in the lung, or indeed of its location within the lung. Unlike VEGFA, PlGF binds exclusively to VEGFR1. Because of this exclusive relationship with VEGFR1, it has been hypothesised that PlGF plays a role in the angiogenic process by displacing VEGFA from the R1 "sink", thereby increasing the fraction of VEGFA available to bind to VEGFR2, the main angiogenic pathway [16]. There is also evidence suggesting that PlGF can form a heterodimer with VEGFA thereby augmenting angiogenesis [17], though this remains controversial [18]. Recent studies have shown that PlGF can cause emphysema when over-expressed in the mouse lung [19], while knocking-out PlGF protected against the development of elastase-induced emphysema [20]. Cheng and colleagues [21] have also reported that PlGF is released from bronchial epithelial cells and potentially contributes to the development of COPD.

The aim of this study was to examine the expression profile of the well-established pro-angiogenic VEGF family members, VEGFA, VEGFB and PlGF, and their receptors, VEGF R1 and VEGF R2, during the development of hypoxic PH. The interactions of VEGFA, VEGFB and PlGF on human pulmonary microvascular endothelial cells were also investigated *in vitro*. The results of this study show that the interactions between the VEGF ligands are complex and critically concentration dependent and may exhibit pro- or anti-angiogenic effects.

## Methods

### Hypoxic Pulmonary Hypertension

All procedures were approved by the University Ethics Committee and conducted under licence from the Department of Health. Adult male specific pathogen free (SPF) Sprague Dawley rats (310-350 g, Harlan, Bicester, UK) were randomly divided between control and hypoxic groups. Animals (n = 8) within the hypoxic groups were exposed to hypoxia (FiO_2_, 0.10) for 1, 3, 7, 14 and 21 days as previously described [4,5,22]. Matched control animals (n = 8) were maintained in the same room under normoxic conditions for the same period of time.

### Surgical Procedures

Following the exposure period, the animals were anesthetised (sodium pentobarbitone 70 mg.kg^-^¹ intra-peritoneal) and anti-coagulated using heparin (1000 I.U/kg intravenously) and then killed by exsanguination [4,5,22]. Following sternotomy, the trachea and pulmonary artery were cannulated and the heart and lungs removed *en bloc*. The right main bronchus, the pulmonary artery and the pulmonary veins were tied with a ligature close to the hilum, the right lung removed and flash frozen.

### Left Lung Preparation and Fixation

Normal saline solution (37°C) was perfused through the pulmonary vasculature via the pulmonary artery until the effluent ran clear. The left lung was then inflated at a standard airway pressure (25 cmH_2_O) with fixative (4% w/v paraformaldehyde in normal saline solution) via the trachea. The pulmonary vessels were perfused with fixative (62.5 cmH_2_O) and the outflow blocked by a clamp around the left atrium. This ensured a standard, constant vascular transmural distending pressure (37.5 cmH_2_O) during fixation [23].

Following introduction of fixative, the major vessels and the airway were tied at the hilum and the lung immersed in fixative for 24 hours and the left lung volume measured by displacement. Following removal of the atria, the right ventricular free wall (RV) was dissected from the left ventricle and septum (LV + S) and each ventricle weighed separately.

### Tissue Preparation

The left lung was processed for stereology and immunohistochemistry as previously described [24]. Briefly, the lung was divided into multiple blocks (approximately 2 × 2 × 4 mm); a systematic randomised sampling strategy was used to select a subset of blocks for embedding in resin and preparation of isotropically uniform random semi-thin (1 micron) sections for stereological analysis. A second subset of blocks was selected using the systemic random sampling strategy for embedding in paraffin wax to prepare sections (5 μm) for immunohistochemistry.

### Stereological Protocol for Assessment of Vascular Parameters

Stereology is a tool that allows quantitative analysis of three dimensional structures to be undertaken using two dimensional sections [24]. The stereological analysis undertaken in this manuscript conforms to the guidelines of the Joint Standards for Quantitative Assessment of Lung Structure, as defined by the American Thoracic Society and European Respiratory Society joint task force [25]. In particular, in all lungs the number of sampled structures of interest (e.g. capillary endothelium, intra-acinar vessels) had a minimum value between 100 and 200, a sampling intensity that ensures precise estimates of the measured value (e.g. surface area, vessel length wall thickness) [26].

Random microscopic images of the lung sections were selected (x20 objective, Olympus BX61 motorised microscope,) using a semi-automated Computer Assisted Stereological Toolbox (CAST) system (Visiopharm integrator system version 2.9.11.0; Olympus Denmark). These images were digitised (Olympus DP70 digital camera) and displayed on screen to allow stereological determination of the length density of the vessels within the gas exchange region of the lung (intra-acinar vessels), lumen diameter and wall thickness, and capillary endothelial surface area as previously described [24,27]. Intra-acinar vessels were identified as those accompanying respiratory bronchioles or more distal airways and alveoli, which had a diameter greater than 10 μm but less than 100 μm. For assessment of capillary endothelial surface area, images of tissue were randomly selected from the tissue section at high magnification (x100 oil immersion objective). All slides were identified by code so that the observer was blinded to the experimental conditions in which the rats had been housed.

### mRNA Extraction and Real-Time PCR Quantification

mRNA was extracted as previously described [28] and the expression of genes of interest was quantified using real-time PCR (TaqMan) performed on duplicate samples. Probe (labelled with FAM/TAMRA) and primer sequences were ordered from ABI as Assays-on-Demand Gene Expression Assays (*Table *1). The Eukaryotic 18S rRNA (VIC/TAMRA) pre-developed assay reagent kit was used as the endogenous control gene according to the TaqMan PCR protocol (Applied Biosystems (ABI), USA). Reactions were carried out on the ABI PRISM 7900 Sequence Detection System and mRNA levels were determined using the standard curve method (ABI Prism 7700 Sequence Detection System User Bulletin #2).

**Table 1:** Real Time PCR TaqMan assays. Real-Time PCR probe and primers name, symbol and ID.

Gene	Symbol	Assay ID
Vascular endothelial growth factor A	Vegfa	Rn00582935_m1
Vascular endothelial growth factor B	Vegfb	Rn01454584_g1
Placenta growth factor	Pgf	Rn00585926_m1
Vascular endothelial growth factor receptor 1	Vegfr1	Rn00570815_m1
Vascular endothelial growth factor receptor 2	Vegfr2	Rn00564986_m1

The above probe/primer pairs were obtained from ABI as Assay on Demand

### Immunohistochemistry and Staining Quantification

Tissue sections from 14-day were cleared of wax in xylene and rehydrated in a series of graded alcohols and immunostained as previously described [29]. The primary antibodies used were anti-human PlGF affinity purified goat IgG (1:20 dilution, final concentration 10 μg/ml, R&D Systems, UK) and anti-human VEGFB affinity purified mouse IgG_1 _(1:10 dilution, final concentration 50 μg/ml, R&D Systems, UK). The appropriate biotin labelled secondary antibodies were used (Biotinylated rabbit anti-goat IgG, 1:50 dilution, Vector Labs, UK or Biotinylated goat anti-mouse IgG, 1:50 dilution, Vector Labs, UK) to detect specific binding using streptavidin-linked horseradish peroxidase and diaminobenzidine (Sigma, Ireland). The volume of tissue stained positively was assessed stereologically by a blinded reviewer as described and expressed as a fraction of the total lung volume.

### Protein Isolation and Western Blot Analysis

Protein was extracted from control and hypoxic right lungs and prepared for western blotting as previously described. Expression of VEGF A protein was assessed using a specific anti-VEGFA antibody (Oncogene). GAPDH was used as an endogenous loading control (Abcam).

### Scratch Assay Protocol

Endothelial scratch assays were carried out as previously described [30]. In brief, human pulmonary microvascular endothelial cells (HMVEC, Lonza, UK) were grown to a confluent monolayer according to the manufacturer's instructions. Following incubation overnight in serum free medium, a single vertical scratch was applied to each monolayer using a sterile pipette tip (S1120-1840, Starlab, UK) and the medium changed to one containing FBS (3%) plus growth factor supplements from which VEGFA had been omitted. After a two-hour "rest" period, the cells were treated with either vehicle (PBS + 0.1% BSA or 35% acetonitrile + 0.1% TFA), or recombinant protein (VEGFA 1-8 ng/ml, PlGF 10-160 ng/ml or VEGFB 20 ng/ml, R&D Systems, UK) as appropriate. Images of the scratch (x10 objective, AxioVision 4.4 software) were used to measure the width of the wound at 0 and 24 hours. Six to nine separate replicates were performed for each experiment and all experiments were performed on cells between Passage 5 and 7.

### Statistical Analysis

All statistics were carried out using Statistica 7.0 software. Results are expressed as mean ± standard error of the mean (±SEM). Data were statistically analysed using a One Way Analysis of Variance (ANOVA) or ANOVA with repeated measures, followed by a Post-hoc Student Newman Keul's test. A value of P < 0.05 was accepted as statistically significant.

## Results

### Haematocrit, Right Ventricular Weight and Left Lung Volume

The mean haematocrit, right ventricular to left ventricle plus septum (RV to LV+S) weight ratios and the left lung volumes of control and hypoxic rats following 14 days exposure to chronic hypoxia are shown in *Table *2. An elevation in haematocrit was observed following hypoxic exposure relative to matched controls, indicating an increase in red blood cell concentration had occurred in response to the hypoxic stimulus. The ratio of RV to LV+S weight was also observed to increase at 14 days of hypoxia relative to matched control values indicating that prolonged chronic hypoxia produced significant RV hypertrophy. Exposure to chronic hypoxia resulted in a significant increase in lung volume at 14 days of hypoxia. These responses developed progressively at 1, 3, 7 and 21 days (data not shown).

**Table 2:** Haematocrit, Cardiac Ventricular Ratios and Left Lung Volume in Control and Hypoxic Conditions

	Control	Hypoxic
**Haematocrit (%)**	44.8 (1.1)	61.0 (0.4) *
**Ventricular Ratio**	0.25 (0.01)	0.4 (0.02) *
**Left Lung Volume (ml)**	3.43 (0.12)	4.14 (0.27) *

Values are mean (±SEM). Ventricular ratio is the ratio between the weight of the right ventricle and the left ventricle plus septum. * indicates a significant difference from matched control (P < 0.05, T-Test)

### Stereological Quantification of Lung Morphology

**Figure 1:**
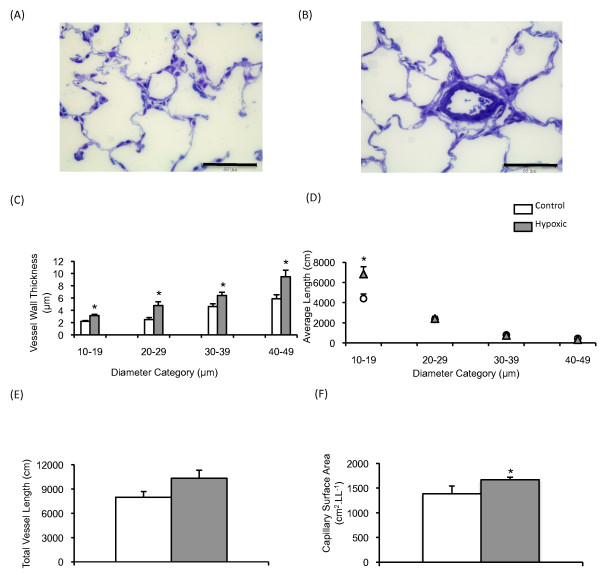
**Stereological analysis of pulmonary vasculature**. Typical images of intra-acinar blood vessels in (A) control and (B) hypoxic lungs. All images are taken from semi-thin (1-2 μm) sections, are stained with Toluidine Blue and were taken with an x40 objective. Intra-acinar blood vessels in normoxic lungs were typically thin walled and had little if any medial layer and an internal elastic lamina only. Intra-acinar blood vessels from hypoxic lungs showed classic vascular remodelling, with the appearance of an external elastic lamina and medial hypertrophy. The scale bar represents 55 μm. (C) Mean wall thickness (μm) of intra-acinar blood vessels (±SEM) following 21 days exposure to control or hypoxic conditions. (D) The average length of intra-acinar blood vessel (cm) per diameter category (±SEM) following 21 days exposure to either normoxic (white circle) or hypoxic (black triangle) conditions. (E) Total intra-acinar blood vessel length was increased in the hypoxic lung, however this increase failed to reach significance (p = 0.08). (F) Mean capillary endothelium surface area (±SEM, cm^2 ^per left lung (LL^-1^)) following 21 days exposure to normoxic or hypoxic conditions. * signifies significant difference from matched control (P < 0.05, ANOVA, post-hoc Student Newman Keuls test). N = 7-8 per group.

*Figure *1 (A*and *1B*) *shows characteristic images of intra-acinar blood vessels within (A) control and (B) hypoxic lungs. An increase in the mean wall thickness was observed in intra-acinar vessels following exposure to 21 days of hypoxia (*figure *1C*)*. A significant increase in the mean length of small (10 μm-20 μm) intra-acinar vessels was also observed when compared to matched controls (*figure *1D*)*. The mean total length of intra-acinar blood vessels in hypoxic lungs was greater than that in control lungs, although this difference was not statistically significant (*figure *1E, *p = 0.08*). Capillary endothelial surface area was significantly increased following 21 days of exposure to hypoxia (*figure *1F).

### Vascular Endothelial Growth Factor (VEGF) Ligands and Receptor mRNA Expression

**Figure 2:**
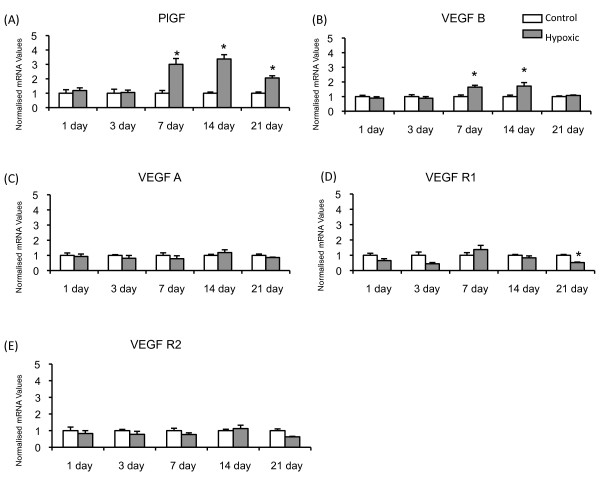
**VEGF Ligand mRNA Expression**. Mean (± SEM) mRNA expression for (A) PlGF, (B) VEGFB, (C) VEGFA, (D) VEGF Receptor 1 and (E) VEGF Receptor 2 in control and hypoxic rat lungs. All values are normalised to matched control group. * signifies significant difference from matched control (P < 0.05, ANOVA, post hoc Student Newman Keuls). N = 6-8 per group.

The most marked change in mRNA expression was observed in PlGF, which was significantly augmented in the hypoxic lung following one week of exposure to chronic hypoxia and this increase in mRNA expression was sustained throughout the exposure period (*figure *2A). VEGFB mRNA was also seen to increase significantly at 7 and 14 days; however, this increase was not as persistent as that of PlGF, as VEGFB mRNA levels were seen to return to baseline at 21 days (*figure *2B). Conversely, exposure to chronic hypoxia did not cause a change in VEGFA mRNA expression at any time point (*figure *2C). VEGF R1 (*figure *2D) expression was significantly decreased following 21 days exposure to chronic hypoxia while VEGF R2 was not altered at any time point examined (*figure *2E).

### PlGF and VEGF B Protein Expression in the Rat Lung

**Figure 3:**
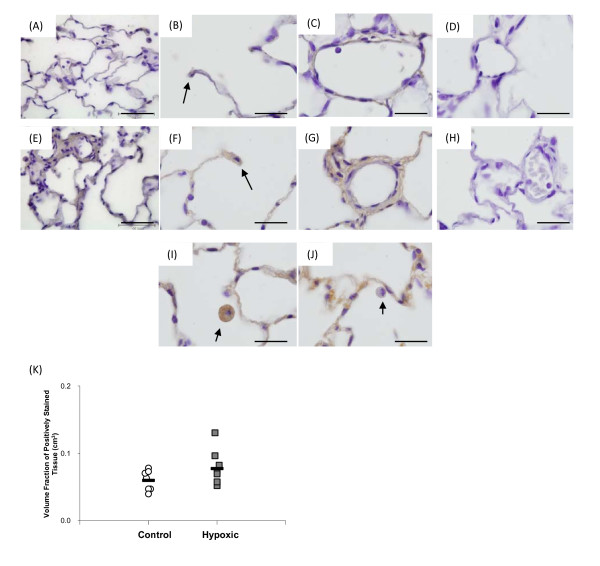
**PlGF protein expression within the rat lung**. Images (A-D) are taken from a normoxic lung, while images (E-J) are from a hypoxic lung. Panels A and E show low magnification images of control and hypoxic tissue. PlGF protein was expressed within the alveolar wall and type II pneumocytes (black arrows, B and F) and the wall of intra-acinar blood vessels (C and G). Panels (I) and (J) show PlGF stained and unstained macrophages from the same hypoxic lung (black arrows). A similar pattern of macrophage staining was observed in control lungs (not shown). Image (D and H) show normoxic (D) and hypoxic (H) lung tissue which was stained with a 1:10 dilution of primary antibody which had been pre-incubated with recombinant PlGF protein. Pre-incubation of the PlGF antibody with recombinant PlGF protein (R&D, UK) successfully blocked staining of the tissue therefore indicating that the staining observed is indeed specific for PlGF protein. All images were taken with an x100 objective, the scale bar represents 20 μm. (K) The volume fraction of PlGF protein was not altered following 14 days exposure to chronic hypoxia compared to its matched normoxic control. The black bar represents the mean value in each group. N = 7-8 per group.

Immunohistochemical analysis was performed following 14 days exposure to chronic hypoxia, the time point at which peak mRNA expression of PlGF and VEGFB were observed. *Figure *3 shows images of PlGF staining in control (*figure *3A-C) and hypoxic (*figure *3E-3G) lungs, with expression being noted in the alveolar wall and type II pneumocytes (*figure *3B and 3F) and the wall of intra-acinar blood vessels (*figure *3C*and *3G). The staining intensity of PlGF was more intense in some hypoxic lungs than in control lungs. However, quantitative stereological analysis of tissue staining positively for PlGF showed that upon exposure to chronic hypoxia the fraction of tissue staining for PlGF did not alter (*figure *3K). PlGF expression was also observed in macrophages, however, it must be noted that not all macrophages stained positively for PlGF under either experimental condition (*figure *3I*and *3J). PlGF was also found in the walls of extra-acinar blood vessels and the epithelium of extra-acinar airways (data not shown).

**Figure 4:**
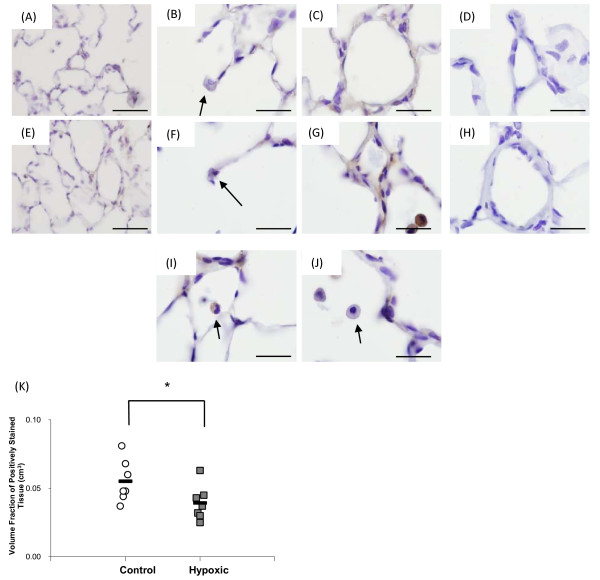
**VEGFB protein expression within the rat lung**. Images (A-D) are taken from a control lung, while images (E-J) are from a hypoxic lung. Panels A and E show low magnification images of control and hypoxic tissue. VEGFB protein was expressed within alveolar wall and type II pneumocytes (black arrows, B and F) and the wall of intra-acinar blood vessels (C and G). Panels (I) and (J) show stained and unstained macrophages from the same hypoxic lung (black arrows). A similar pattern of macrophage staining was observed in control lungs (not shown). Images (D) and (H) show control (D) and hypoxic (H) lung tissue to which Isotype matched IgG (mouse IgG_1, _R&D, UK) was added to the slide instead of primary antibody. No staining was observed in the IgG slides, therefore indicating that the staining observed is indeed specific for VEGFB protein. All images were taken with an x100 objective, the scale bar represents 20 μm. (K) The volume fraction of VEGFB protein was significantly decreased following 14 days exposure to chronic hypoxia compared to its matched normoxic control. The black bar represents the mean value in each group. * signifies significant difference from matched control (p < 0.05, T-Test, post-hoc Mann Whitney U). N = 7 per group.

VEGFB protein was basally expressed in the lung. *Figure *4 shows images of VEGFB staining in control (*figure *4A-4C) and hypoxic (*figure *4E-4G) lungs, with expression being noted in the alveolar wall and type II pneumocytes (*figure *4B*and *4F) and the wall of intra-acinar blood vessels (*figure *4C*and *4G). No difference in the staining intensity of VEGFB was observed in the hypoxic lung. However, stereological analysis of the volume fraction of positively stained VEGFB tissue in the lung revealed that upon exposure to chronic hypoxia, the volume fraction of VEGFB stained cells was significantly decreased compared to normoxic control (*figure *4K), suggesting reduced total VEGFB expression within the lung. As with PlGF, expression of VEGFB was observed in macrophages, however, not all macrophages stained positively for VEGFB under either control or hypoxic conditions (*figure *4I*and *4J). VEGFB was also found in the wall of extra-acinar blood vessels and the epithelial layer of extra-acinar airways (data not shown).

### VEGFA protein expression

**Figure 5:**
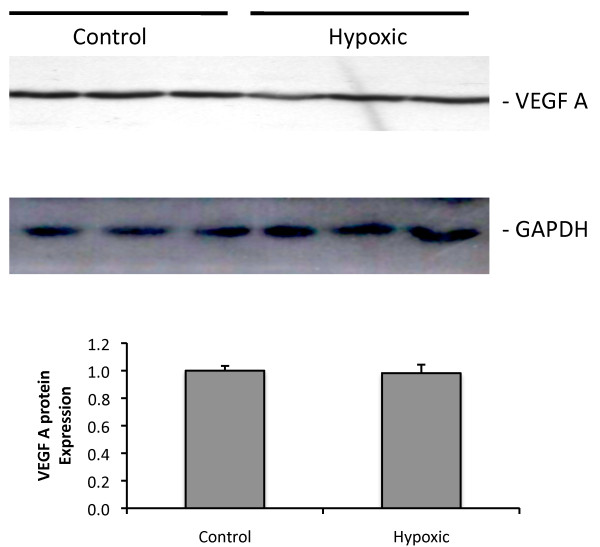
**VEGFA protein expression in control and hypoxic rat lung**. Characteristic western blots for VEGFA and GAPDH. There were no alterations in protein expression of the VEGF ligand in the hypoxic lung. GAPDH served as a loading control. N = 3 per group.

Western blotting showed that VEGFA protein expression was not altered in response following 14 days exposure to chronic hypoxia (*figure *5).

### Role of VEGF Ligands in Endothelial Cell Wound Healing

We next examined the effects of PlGF and VEGFB on pulmonary microvascular endothelial cell regeneration and repair using a scratch assay in endothelial cell monolayers. Since VEGFA is constitutively expressed in the lung and was unchanged in hypoxia, we compared responses to both PlGF and VEGFB to those produced by VEGFA and the interactions of these ligands with VEGFA.

**Figure 6:**
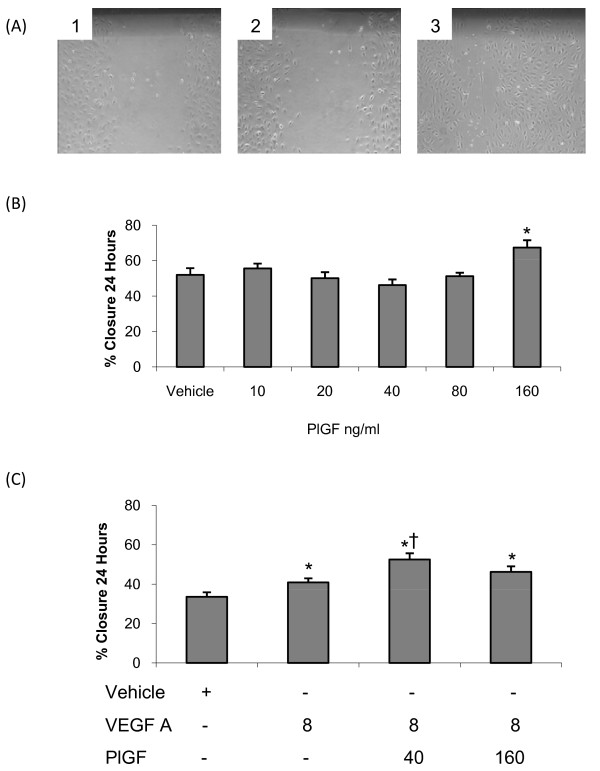
**The actions of PlGF on endothelial cell wound healing**. (A) Human pulmonary microvascular endothelial cell scratch assay in the presence of vehicle (panel 1), PlGF (40 ng/ml; panel 2) and PlGF (160 ng/ml; panel 3). (B) Mean (±SEM) percentage wound closure over a 24-hour period in the presence of PlGF under normoxic conditions. (C) Mean (±SEM) percentage wound closure over a 24-hour period in the presence of VEGFA (8 ng/ml)-PlGF (40 ng/ml) and VEGFA (8 ng/ml)-PlGF (160 ng/ml) in normoxic human pulmonary microvascular endothelial cells. * signifies a significant increase in wound healing compared to vehicle, † signifies significant difference from VEGFA alone (8 ng/ml) (P < 0.05, ANOVA, post hoc Student Newman Keuls). N = 6-9 per group.

Representative images of normoxic wound healing assays in the presence of varying concentrations of recombinant PlGF protein (panels 1-3; vehicle, 40 ng/ml and 160 ng/ml respectively) are shown in *figure *6A. Increasing concentrations of PlGF (10-80 ng/ml) did not alter the rate of human microvascular endothelial cell wound closure compared to vehicle. However, the highest concentration of PlGF tested (160 ng/ml) significantly increased the rate of wound healing when compared to vehicle (*figure *6B).

When administered with VEGFA (8 ng/ml), PlGF exerted concentration dependent reponses. A low concentration of PlGF (40 ng/ml) in combination with VEGFA significantly increased the rate of wound closure compared to VEGFA alone (*figure *6C). However, addition of a higher concentration of PlGF (160 ng/ml) to VEGFA did not significantly increase the rate of wound closure compared to VEGFA alone (*figure *6C).

**Figure 7:**
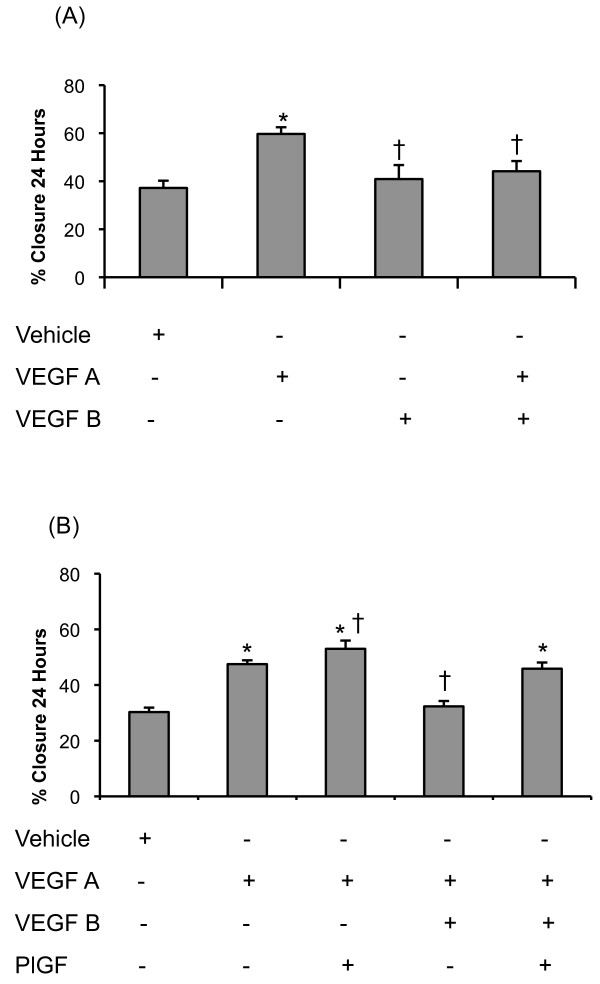
**VEGF ligand interactions in endothelial cell wound healing**. (A) Mean (±SEM) percentage wound closure over a 24-hour period in the presence of VEGFA (8 ng/ml), VEGFB (20 ng/ml) and VEGFA (8 ng/ml)-VEGFB (20 ng/ml). (B) Mean (±SEM) percentage wound closure over a 24-hour period in the presence of VEGFA (8 ng/ml), PlGF (40 ng/ml) and VEGFB (20 ng/ml) in normoxic human pulmonary microvascular endothelial cells. * signifies a significant increase in wound healing compared to vehicle, † signifies significant difference from VEGFA (8 ng/ml) (P < 0.05, ANOVA, post hoc Student Newman Keuls). N= 6 per group.

VEGFB alone did not augment the rate of wound healing over vehicle. However, VEGFB inhibited the actions of VEGFA (*figure *7A). When PlGF was added to VEGFA and VEGFB, wound healing was not significantly different from VEGFA alone; that is PlGF antagonised the inhibitory effects of VEGFB (*figure *7B).

## Discussion

We report here for the first time changes in PlGF and VEGFB expression in the lung in response to hypoxia *in vivo *mimicking that found in lung diseases. In addition we show that VEGFB can inhibit the actions of VEGFA on pulmonary microvascular endothelial regeneration and repair. Furthermore, PlGF can counteract the inhibitory effects of VEGFB.

The chronically hypoxic rats reported here showed the well-documented consequences of such exposure including right ventricular hypertrophy, elevated haematocrit and increased lung volumes (Table 2), as previously reported [4,5,28,31-37]. In addition, we observed increased total length of intra-acinar vessels within the smallest diameter category (10-20 μm diameter) and capillary endothelial surface area (indices of angiogenesis) that are in keeping with previous reports [4,5,28,38-40]. The increase in total intra-acinar vessel length was not statistically significant (P = 0.08), raising the possibility that the increase in length of small intra-acinar vessels may have occurred as a result of vessels "migrating" from larger diameter categories due to wall remodeling. However, when considered together with the evidence of capillary angiogenesis in these lungs and our previous demonstrations of increased intra-acinar vessel length in response to hypoxia, the data are consistent with new vessel growth [4,5,28]. Characteristic remodelling of the pulmonary vascular walls was also observed (*figure *1).

Expression of both PlGF and VEGFB mRNA increased in the hypoxic lung at later time points which corresponded to times at which capillary angiogenesis is observed in the hypoxic lung [4,5]. Immunohistochemistry showed that PlGF protein was expressed in the normal lung basally and that, although intensity of staining was increased in some hypoxic lungs, this was not a consistent finding. Moreover, the proportion of cells expressing PlGF did not change upon exposure to chronic hypoxia. Thus there was not a clear increase in PlGF protein despite the increase in mRNA. Conversely, immunohistochemical analysis of VEGFB showed that the proportion of cells expressing this protein was significantly reduced in the hypoxic lung (*figure *4), suggesting a reduction in VEGFB protein within the alveolar wall despite increased VEGFB mRNA.

The finding that VEGFA mRNA expression was not altered (*figure *2) is similar to those of many previous reports of unaltered mRNA expression [4,36,41-43], although other reports suggest that VEGFA mRNA expression is augmented [40,44,45] or VEGFA mRNA is decreased following 14 days exposure to chronic hypoxia [46]. Similarly VEGFA protein expression has been reported as augmented [40,44,45], reduced [46] or unchanged [47]. We found that VEGFA protein expression was not altered following 14 days exposure to chronic hypoxia (*figure *5), a finding similar to that of Engebretsen and colleagues [47]. These apparently discrepant results may arise due to differences in experimental protocols, durations of exposure and species or strain differences (e.g. see [47]). Differences in antibodies used may also contribute to the different reports; given the very extensive homology between different members of the VEGF family ligands and their many splice variants, different antibodies may be detecting different proteins from within this large group. The VEGFA antibody that we used identified a single band on western blots of rat lungs at a molecular weight compatible with homodimeric VEGFA165.

Expression of VEGFR1 and VEGFR2 mRNA did not change substantially in response to hypoxia. However, it should be noted that the soluble form of VEGFR1, sVEGFR1 or s-Flt1 may also have been detected by the TaqMan assay used in this experiment and further work is required to determine the precise expression of each individually.

Within the systemic circulation, VEGFA is consistently reported to increase in response to exposure to chronic hypoxia [48-50], while also being upregulated in cancer [51-53]. It may at first appear surprising that the well known pro-angiogenic ligand VEGFA was not observed to increase in the hypoxic lung given the well characterised increases in its expression within the hypoxic systemic circulation. It is important to note that tumours and ischemic tissues have a PO_2 _that is much lower than the PO_2 _that would ever be encountered within the hypoxic alveoli. For example, the PO_2 _of the alveoli during the development of hypoxic pulmonary vasoconstriction and pulmonary hypertension is 5.0-8.0 kPa, a value much higher than that of a systemic organ under conditions of normal oxygenation [54-56]. It is therefore likely that the mechanisms controlling gene expression in the two separate circulatory systems are different [57]. The different control mechanisms and PO_2 _could account for the differing expression profiles encountered in the pulmonary and systemic circulations.

For both PlGF and VEGFB, the changes in protein and mRNA expression were discordant i.e. mRNA of both was increased while protein expression remained unchanged (PlGF) or was reduced (VEGFB). Such discordance is well recognized in other organs e.g. following the onset of skeletal muscle ischaemia VEGF mRNA is initially elevated but subsequently falls below normoxic values while VEGF protein expression increases [58]. Differences in mRNA and protein behaviour can arise by a number of mechanisms e.g. changes in the rate of translation or modification of the rate of protein breakdown through post-translational mechanisms.

To gain insight into the functional significance of VEGFA, VEGFB and PlGF expression, wound healing assay experiments were conducted *in vitro *on human pulmonary microvascular endothelial cells. Although no changes in the expression profile of VEGFA were observed, VEGFA is constitutively expressed in the lung; therefore the actions of PlGF and VEGFB were examined both in the presence and the absence of VEGFA. The concentration used (8 ng/ml) was chosen as typical of that required for survival of pulmonary microvascular endothelial cells in culture. At the lower concentrations examined (10-80 ng/ml), PlGF did not stimulate wound healing, a finding similar to those of Cao et al [59] and Carmeliet et al [60]. However, the highest PlGF concentration tested stimulated wound healing effectively (*figure *6A*and *5B). Given the expression of VEGFA in the lung, the actions of PlGF in the presence of VEGFA were also examined and showed that PlGF at a low concentration augmented wound healing compared to VEGFA alone (*figure *6C), a synergistic effect that has not previously been recognized. However, when PlGF was administered at higher concentrations, wound healing was not increased compared to VEGFA alone (*figure *6C) i.e. the synergistic effect was lost. In contrast, these high concentrations of PlGF when used in the absence of VEGFA increased the rate of wound healing. Thus the effects of these two proteins depend critically on their relative concentrations.

Further insights into the relationship between VEGFA and PlGF can be obtained by comparing the results of this study with those of Carmeliet et al [60]. That study involved administering VEGFA and PlGF to wild-type capillary endothelial cells and PlGF-/- capillary endothelial cells. They observed that when administered alone to PlGF-/- cells, the mitogenic and proliferative actions of VEGFA were greatly reduced compared to wild-type results. However, when VEGFA was co-administered with recombinant PlGF to PlGF-/- cells, the degree of migration and proliferation was similar to wild-type cells in the presence of VEGFA alone. Therefore, Carmeliet and colleagues surmised that the actions of VEGF-A are as a result of a degree of synergism with endogenous PlGF within capillary endothelial cells. The present finding extends the results of Carmeliet and colleagues by showing that a critical low concentration of PlGF is required for optimal VEGFA activity, whereas at higher concentrations this potentiating action is lost.

Our findings provide a potential explanation for the previously puzzling finding that mice over-expressing PlGF developed emphysema [19]. It is well established that loss of VEGFA activity in the lung can result in the development of emphysema [61]. Excess PlGF expression in the lung of the transgenic mouse could cause a loss of synergism between PlGF and VEGFA, similar to that demonstrated here *in vitro*, resulting in decreased VEGFA activity. Furthermore, these findings suggest that the increased PlGF expression observed in the serum and BAL fluid of COPD patients could contribute to the progression of emphysema and COPD [21]. While attractive, this hypothesis will require direct experimental verification.

Surprisingly, VEGFB was observed to inhibit the wound healing actions of VEGFA, while having little effect on its own (*figure *7A). It suggests that the reduction in VEGFB protein that we observed in the lung *in vivo *would have augmented VEGFA actions and thus contributed to hypoxia-induced capillary angiogenesis. To date the role of VEGFB within the lung has not been fully elucidated. Investigations of the systemic circulation employing a VEGFB knockout mouse model showed evidence of coronary vasculature dysfunction [62] but no report of any abnormalities within the pulmonary circulation. Further studies have shown a role for VEGFB as a pro-angiogenic mediator in arthritis induced synovial angiogenesis [63]. However, none of those investigations examined the interactions of other VEGF ligands with VEGFB.

Since VEGFA, VEGFB and PlGF are all expressed together, it was of interest to examine the combined effects of these ligands acting simultaneously. The finding that PlGF and VEGFB antagonise each other when administered with VEGF-A is an interesting novel finding that emphasises the complex interactions of the VEGF ligands in the pulmonary circulation (*figure *7B).

## Conclusions

The pro-angiogenic ligands of the VEGF family (VEGFA, VEGFB and PlGF) have an important homeostatic role to play within the pulmonary circulation. The findings reported here demonstrate their differing temporal expression patterns within the hypoxic lung, and show that they can potentially act as inhibitors of angiogenic behaviour in pulmonary endothelial cells. Thus their actions *in vivo *will depend on their specific concentrations within the microenvironment of the alveolar wall during the course of adaptation to pulmonary hypoxia.

## Competing interests

The authors declare that they have no competing interests.

## Authors' contributions

MS carried out the animal studies, real time PCR, immunohistochemistry, scratch assay and statistical analysis. KH performed the western blots. KH and CMC participated in the design of the study, MS and PMcL conceived of the study, participated in its design and coordination and drafted the manuscript. All authors read and approved the final manuscript.

## References

[B1] MacNee W Pathophysiology of cor pulmonale in chronic obstructive pulmonary disease. Part two Am J Respir Crit Care Med 1994 150 4 1158 68 7921453 10.1164/ajrccm.150.4.7921453

[B2] MacNee W Pathophysiology of cor pulmonale in chronic obstructive pulmonary disease. Part One Am J Respir Crit Care Med 1994 150 3 833 52 8087359 10.1164/ajrccm.150.3.8087359

[B3] Mandegar M Cellular and molecular mechanisms of pulmonary vascular remodeling: role in the development of pulmonary hypertension Microvasc Res 2004 68 2 75 103 10.1016/j.mvr.2004.06.001 15313118

[B4] Hyvelin JM Inhibition of Rho-kinase attenuates hypoxia-induced angiogenesis in the pulmonary circulation Circ Res 2005 97 2 185 91 10.1161/01.RES.0000174287.17953.83 15961717

[B5] Howell K Preston RJ McLoughlin P Chronic hypoxia causes angiogenesis in addition to remodelling in the adult rat pulmonary circulation J Physiol 2003 547 Pt 1 133 45 10.1113/jphysiol.2002.030676 12562951 PMC2342608

[B6] Le Cras TD Treatment of newborn rats with a VEGF receptor inhibitor causes pulmonary hypertension and abnormal lung structure Am J Physiol Lung Cell Mol Physiol 2002 283 3 L555 62 12169575 10.1152/ajplung.00408.2001

[B7] Oka M Rho kinase-mediated vasoconstriction is important in severe occlusive pulmonary arterial hypertension in rats Circ Res 2007 100 6 923 9 10.1161/01.RES.0000261658.12024.18 17332430

[B8] Voelkel NF Janus face of vascular endothelial growth factor: the obligatory survival factor for lung vascular endothelium controls precapillary artery remodeling in severe pulmonary hypertension Crit Care Med 2002 30 5 Suppl S251 6 10.1097/00003246-200205001-00013 12004244

[B9] Partovian C Adenovirus-mediated lung vascular endothelial growth factor overexpression protects against hypoxic pulmonary hypertension in rats Am J Respir Cell Mol Biol 2000 23 6 762 71 11104729 10.1165/ajrcmb.23.6.4106

[B10] Byrne AM Bouchier-Hayes DJ Harmey JH Angiogenic and cell survival functions of vascular endothelial growth factor (VEGF) J Cell Mol Med 2005 9 4 777 94 10.1111/j.1582-4934.2005.tb00379.x 16364190 PMC6740098

[B11] Wirzenius M Distinct vascular endothelial growth factor signals for lymphatic vessel enlargement and sprouting J Exp Med 2007 204 6 1431 40 10.1084/jem.20062642 17535974 PMC2118625

[B12] Ueda N Pseudocowpox virus encodes a homolog of vascular endothelial growth factor Virology 2003 305 2 298 309 10.1006/viro.2002.1750 12573575

[B13] Staton CA Neuropilins in physiological and pathological angiogenesis J Pathol 2007 212 3 237 48 10.1002/path.2182 17503412

[B14] Harper SJ Bates DO VEGF-A splicing: the key to anti-angiogenic therapeutics? Nat Rev Cancer 2008 8 11 880 7 10.1038/nrc2505 18923433 PMC2613352

[B15] Varet J VEGF in the lung: a role for novel isoforms Am J Physiol Lung Cell Mol Physiol 2010 298 6 L768 74 10.1152/ajplung.00353.2009 20228180 PMC2886605

[B16] Park JE Placenta growth factor. Potentiation of vascular endothelial growth factor bioactivity, in vitro and in vivo, and high affinity binding to Flt-1 but not to Flk-1/KDR J Biol Chem 1994 269 41 25646 54 7929268

[B17] DiSalvo J Purification and characterization of a naturally occurring vascular endothelial growth factor.placenta growth factor heterodimer J Biol Chem 1995 270 13 7717 23 10.1074/jbc.270.13.7717 7706320

[B18] Eriksson A Placenta growth factor-1 antagonizes VEGF-induced angiogenesis and tumor growth by the formation of functionally inactive PlGF-1/VEGF heterodimers Cancer Cell 2002 1 1 99 108 10.1016/S1535-6108(02)00028-4 12086892

[B19] Tsao PN Overexpression of placenta growth factor contributes to the pathogenesis of pulmonary emphysema Am J Respir Crit Care Med 2004 169 4 505 11 10.1164/rccm.200306-774OC 14644931

[B20] Cheng SL Prevention of elastase-induced emphysema in placenta growth factor knock-out mice Respir Res 2009 10 115 10.1186/1465-9921-10-115 19930612 PMC2789728

[B21] Cheng SL Increased expression of placenta growth factor in COPD Thorax 2008 63 6 500 6 10.1136/thx.2007.087155 18202163 PMC2571977

[B22] Ooi H Chronic hypercapnia inhibits hypoxic pulmonary vascular remodeling Am J Physiol Heart Circ Physiol 2000 278 2 H331 8 10666061 10.1152/ajpheart.2000.278.2.H331

[B23] Tsukimoto K Ultrastructural appearances of pulmonary capillaries at high transmural pressures J Appl Physiol 1991 71 2 573 82 1718936 10.1152/jappl.1991.71.2.573

[B24] Bolender RP Hyde DM Dehoff RT Lung morphometry: a new generation of tools and experiments for organ, tissue, cell, and molecular biology Am J Physiol 1993 265 6 Pt 1 L521 48 8279570 10.1152/ajplung.1993.265.6.L521

[B25] Hsia CC An official research policy statement of the American Thoracic Society/European Respiratory Society: standards for quantitative assessment of lung structure Am J Respir Crit Care Med 2010 181 4 394 418 10.1164/rccm.200809-1522ST 20130146 PMC5455840

[B26] Gundersen HJ The efficiency of systematic sampling in stereology--reconsidered J Microsc 1999 193 Pt 3 199 211 10.1046/j.1365-2818.1999.00457.x 10348656

[B27] Howell K Hopkins N McLoughlin P Combined confocal microscopy and stereology: a highly efficient and unbiased approach to quantitative structural measurement in tissues Exp Physiol 2002 87 6 747 56 10.1113/eph8702477 12530405

[B28] Howell K L-Arginine promotes angiogenesis in the chronically hypoxic lung: a novel mechanism ameliorating pulmonary hypertension Am J Physiol Lung Cell Mol Physiol 2009 296 6 L1042 50 10.1152/ajplung.90327.2008 19346433

[B29] Cadogan E Enhanced expression of inducible nitric oxide synthase without vasodilator effect in chronically infected lungs Am J Physiol 1999 277 3 Pt 1 L616 27 10484470 10.1152/ajplung.1999.277.3.L616

[B30] Costello CM Lung-selective gene responses to alveolar hypoxia: potential role for the bone morphogenetic antagonist gremlin in pulmonary hypertension Am J Physiol Lung Cell Mol Physiol 2008 295 2 L272 84 10.1152/ajplung.00358.2007 18469115

[B31] Cunningham EL Brody JS Jain BP Lung growth induced by hypoxia J Appl Physiol 1974 37 3 362 6 4414528 10.1152/jappl.1974.37.3.362

[B32] Kay JM Suyama KL Keane PM Failure to show decrease in small pulmonary blood vessels in rats with experimental pulmonary hypertension Thorax 1982 37 12 927 30 10.1136/thx.37.12.927 6820578 PMC459460

[B33] Mehta S Short-term pulmonary vasodilation with L-arginine in pulmonary hypertension Circulation 1995 92 6 1539 45 7664438 10.1161/01.cir.92.6.1539

[B34] Mitani Y Maruyama K Sakurai M Prolonged administration of L-arginine ameliorates chronic pulmonary hypertension and pulmonary vascular remodeling in rats Circulation 1997 96 2 689 97 9244244

[B35] Nagaoka T Inhaled Rho kinase inhibitors are potent and selective vasodilators in rat pulmonary hypertension Am J Respir Crit Care Med 2005 171 5 494 9 10.1164/rccm.200405-637OC 15563635

[B36] Partovian C Heart and lung VEGF mRNA expression in rats with monocrotaline- or hypoxia-induced pulmonary hypertension Am J Physiol 1998 275 6 Pt 2 H1948 56 9843792 10.1152/ajpheart.1998.275.6.H1948

[B37] Rabinovitch M Rat pulmonary circulation after chronic hypoxia: hemodynamic and structural features Am J Physiol 1979 236 6 H818 27 443445 10.1152/ajpheart.1979.236.6.H818

[B38] Pascaud MA Lung overexpression of angiostatin aggravates pulmonary hypertension in chronically hypoxic mice Am J Respir Cell Mol Biol 2003 29 4 449 57 10.1165/rcmb.2002-0120OC 12714372

[B39] Yamaji-Kegan K Hypoxia-induced mitogenic factor has proangiogenic and proinflammatory effects in the lung via VEGF and VEGF receptor-2 Am J Physiol Lung Cell Mol Physiol 2006 291 6 L1159 68 10.1152/ajplung.00168.2006 16891392

[B40] Yamaji-Kegan K IL-4 is proangiogenic in the lung under hypoxic conditions J Immunol 2009 182 9 5469 76 10.4049/jimmunol.0713347 19380795 PMC10204605

[B41] Berg JT Alveolar hypoxia increases gene expression of extracellular matrix proteins and platelet-derived growth factor-B in lung parenchyma Am J Respir Crit Care Med 1998 158 6 1920 8 9847287 10.1164/ajrccm.158.6.9804076

[B42] Kugathasan L Role of angiopoietin-1 in experimental and human pulmonary arterial hypertension Chest 2005 128 6 Suppl 633S 642S 10.1378/chest.128.6_suppl.633S 16373885

[B43] Pfeifer M Vascular remodeling and growth factor gene expression in the rat lung during hypoxia Respir Physiol 1998 111 2 201 12 10.1016/S0034-5687(97)00102-3 9574871

[B44] Christou H Increased vascular endothelial growth factor production in the lungs of rats with hypoxia-induced pulmonary hypertension Am J Respir Cell Mol Biol 1998 18 6 768 76 9618381 10.1165/ajrcmb.18.6.2980

[B45] Tuder RM Flook BE Voelkel NF Increased gene expression for VEGF and the VEGF receptors KDR/Flk and Flt in lungs exposed to acute or to chronic hypoxia. Modulation of gene expression by nitric oxide J Clin Invest 1995 95 4 1798 807 10.1172/JCI117858 7706486 PMC295709

[B46] Yamamoto A Downregulation of angiopoietin-1 and Tie2 in chronic hypoxic pulmonary hypertension Respiration 2008 75 3 328 38 10.1159/000112432 18073453

[B47] Engebretsen BJ Acute hypobaric hypoxia (5486 m) induces greater pulmonary HIF-1 activation in hilltop compared to madison rats High Alt Med Biol 2007 8 4 312 21 10.1089/ham.2007.1031 18081507

[B48] Li H Hypoxia-induced increase in soluble Flt-1 production correlates with enhanced oxidative stress in trophoblast cells from the human placenta Placenta 2005 26 2-3 210 7 10.1016/j.placenta.2004.05.004 15708122

[B49] Sivakumar V Vascular endothelial growth factor and nitric oxide production in response to hypoxia in the choroid plexus in neonatal brain Brain Pathol 2008 18 1 71 85 10.1111/j.1750-3639.2007.00104.x 17924979 PMC8095499

[B50] Xu F Severinghaus JW Rat brain VEGF expression in alveolar hypoxia: possible role in high-altitude cerebral edema J Appl Physiol 1998 85 1 53 7 9655755 10.1152/jappl.1998.85.1.53

[B51] Fechner G Evaluation of hypoxia-mediated growth factors in a novel bladder cancer animal model Anticancer Res 2007 27 6B 4225 31 18225594

[B52] Itakura E Detection and characterization of vascular endothelial growth factors and their receptors in a series of angiosarcomas J Surg Oncol 2008 97 1 74 81 10.1002/jso.20766 18041747

[B53] Shimoda K Vascular endothelial growth factor/vascular permeability factor mRNA expression in patients with chronic hepatitis C and hepatocellular carcinoma Int J Oncol 1999 14 2 353 9 9917513 10.3892/ijo.14.2.353

[B54] Tsai AG Microvascular and tissue oxygen gradients in the rat mesentery Proc Natl Acad Sci USA 1998 95 12 6590 5 10.1073/pnas.95.12.6590 9618456 PMC22570

[B55] Leong CL Evidence that renal arterial-venous oxygen shunting contributes to dynamic regulation of renal oxygenation Am J Physiol Renal Physiol 2007 292 6 F1726 33 10.1152/ajprenal.00436.2006 17327497

[B56] Archer SL Preferential expression and function of voltage-gated, O2-sensitive K+ channels in resistance pulmonary arteries explains regional heterogeneity in hypoxic pulmonary vasoconstriction: ionic diversity in smooth muscle cells Circ Res 2004 95 3 308 18 10.1161/01.RES.0000137173.42723.fb 15217912

[B57] Leonard MO Hypoxia selectively activates the CREB family of transcription factors in the in vivo lung Am J Respir Crit Care Med 2008 178 9 977 83 10.1164/rccm.200712-1890OC 18689465 PMC2643223

[B58] Milkiewicz M Vascular endothelial growth factor mRNA and protein do not change in parallel during non-inflammatory skeletal muscle ischaemia in rat J Physiol 2006 577 Pt 2 671 8 10.1113/jphysiol.2006.113357 16990404 PMC1890445

[B59] Cao Y Heterodimers of placenta growth factor/vascular endothelial growth factor. Endothelial activity, tumor cell expression, and high affinity binding to Flk-1/KDR J Biol Chem 1996 271 6 3154 62 10.1074/jbc.271.6.3154 8621715

[B60] Carmeliet P Synergism between vascular endothelial growth factor and placental growth factor contributes to angiogenesis and plasma extravasation in pathological conditions Nat Med 2001 7 5 575 83 10.1038/87904 11329059

[B61] Kasahara Y Inhibition of VEGF receptors causes lung cell apoptosis and emphysema J Clin Invest 2000 106 11 1311 9 10.1172/JCI10259 11104784 PMC387249

[B62] Bellomo D Mice lacking the vascular endothelial growth factor-B gene (Vegfb) have smaller hearts, dysfunctional coronary vasculature, and impaired recovery from cardiac ischemia Circ Res 2000 86 2 E29 35 10666423 10.1161/01.res.86.2.e29

[B63] Mould AW Vegfb gene knockout mice display reduced pathology and synovial angiogenesis in both antigen-induced and collagen-induced models of arthritis Arthritis Rheum 2003 48 9 2660 9 10.1002/art.11232 13130487

